# Extracellular DNA facilitates bacterial adhesion during *Burkholderia pseudomallei* biofilm formation

**DOI:** 10.1371/journal.pone.0213288

**Published:** 2019-03-11

**Authors:** Rattiyaphorn Pakkulnan, Chitchanok Anutrakunchai, Sakawrat Kanthawong, Suwimol Taweechaisupapong, Pisit Chareonsudjai, Sorujsiri Chareonsudjai

**Affiliations:** 1 Department of Microbiology, Faculty of Medicine, Khon Kaen University, Khon Kaen, Thailand; 2 Melioidosis Research Center, Khon Kaen University, Khon Kaen, Thailand; 3 Biofilm Research Group, Khon Kaen University, Khon Kaen, Thailand; 4 Department of Oral Diagnosis, Faculty of Dentistry, Khon Kaen University, Khon Kaen, Thailand; 5 Department of Environmental Science, Faculty of Science, Khon Kaen University, Khon Kaen, Thailand; University of Toledo College of Medicine and Life Sciences, UNITED STATES

## Abstract

The biofilm-forming ability of *Burkholderia pseudomallei* is crucial for its survival in unsuitable environments and is correlated with antibiotic resistance and relapsing cases of melioidosis. Extracellular DNA (eDNA) is an essential component for biofilm development and maturation in many bacteria. The aim of this study was to investigate the eDNA released by *B*. *pseudomallei* during biofilm formation using DNase treatment. The extent of biofilm formation and quantity of eDNA were assessed by crystal-violet staining and fluorescent dye-based quantification, respectively, and visualized by confocal laser scanning microscopy (CLSM). Variation in *B*. *pseudomallei* biofilm formation and eDNA quantity was demonstrated among isolates. CLSM images of biofilms stained with FITC-ConA (biofilm) and TOTO-3 (eDNA) revealed the localization of eDNA in the biofilm matrix. A positive correlation of biofilm biomass with quantity of eDNA during the 2-day biofilm-formation observation period was found. The increasing eDNA quantity over time, despite constant living/dead ratios of bacterial cells during the experiment suggests that eDNA is delivered from living bacterial cells. CLSM images demonstrated that depletion of eDNA by DNase I significantly lessened bacterial attachment (if DNase added at 0 h) and biofilm developing stages (if added at 24 h) but had no effect on mature biofilm (if added at 45 h). Collectively, our results reveal that eDNA is released from living *B*. *pseudomallei* and is correlated with biofilm formation. It was also apparent that eDNA is essential during bacterial cell attachment and biofilm-forming steps. The depletion of eDNA by DNase may provide an option for the prevention or dispersal of *B*. *pseudomallei* biofilm.

## Introduction

Biofilm provides shelter for various pathogens and its formation is clearly essential for microbial survival in diverse environments, potentially leading to increased virulence [1, 2]. The microorganisms in biofilms are encased in a hydrated extracellular matrix of biopolymers, mainly polysaccharides, proteins, lipids and extracellular DNA (eDNA) [3]. These various components provide biofilm stabilization, ability to adhere to surfaces, and resistance to harmful effects of antimicrobial agents and host immune responses [3, 4]. Eradication of bacteria in biofilms is difficult and much attention has focused either on biofilm matrix disruption to improve antibiotic perception or on ways to switch the bacteria to the more vulnerable planktonic state.

eDNA is a crucial component of biofilms particularly for initial attachment and in the early stages of bacterial biofilm formation [5–15]. Evidence for the role of eDNA has come from *Pseudomonas aeruginosa* biofilm treated with DNase I and suggested that this enzyme might be useful as a prophylactic to prevent biofilm formation in cystic fibrosis lung patients [10]. Moreover, eDNA not only acidifies *P*. *aeruginosa* biofilm but also shields biofilm from aminoglycosides and antimicrobial peptides [11]. In the case of a food-borne pathogen, *Listeria monocytogenes*, eDNA is a key component of the biofilm matrix during both initial attachment and early biofilm formation. Enzymatic disruption using DNase can be targeted for prevention and removal of the biofilms [5, 9, 15]. Additionally, eDNA of *Helicobacter pylori* was demonstrated as a key component of biofilm matrix [12]. In *Staphylococcus aureus*, eDNA has a crucial role in biofilm attachment and acts as an electrostatic net that tethers cells together inside the biofilm matrix [13, 14]. However, the role of eDNA during biofilm formation of *Neisseria meningitidis* appears to vary among strains [7].

*Burkholderia pseudomallei*, a Gram-negative environmental bacterium, is a causative agent of melioidosis first discovered in 1911 [16]. Since then, the global burden of life-threatening melioidosis has been increasing, being as high as 165,000 cases per year of which an estimated 89,000 (54%) are fatal [17, 18]. Precise diagnostic tests with timely and effective therapeutic approaches are crucial to improve patients’ outcomes and to lower the mortality rate [17]. However, there is a high relapse rate of melioidosis in endemic areas due to failure to clear an infection completely [19–23]. Relapse correlates with biofilm formation by *B*. *pseudomallei* [21]. Biofilm formed *by B*. *pseudomallei* likely limits antibiotic penetration, leading to resistance to conventional antibiotics such as doxycycline, ceftazidime, imipenem, and trimethoprim-sulfamethoxazole [24–26]. In addition, *B*. *pseudomallei* biofilm contributes to initial attachment to human lung epithelial cells, leading to apoptosis of host cells and proinflammatory responses [27].

There has been little research on the role of eDNA in *B*. *pseudomallei* biofilms. Austin and colleagues [28] noticed an accumulation of *B*. *pseudomallei* eDNA for the duration of stomach colonization in an *in vitro* stomach model. Meanwhile, *B*. *thailandensis*, a closely related species, required eDNA for biofilm formation stage but not for the initial attachment stage [29]. The role of eDNA during *B*. *pseudomallei* biofilm development therefore needs to be clarified. The aim of our study was therefore to demonstrate the role of eDNA during three stages of *B*. *pseudomallei* biofilm development: initial attachment of cells to the surface, early development of biofilm architecture and maturation of biofilm architecture [30] using DNase I treatment. The information gained from this study highlights the role of *B*. *pseudomallei* eDNA during biofilm formation. Consequently, biofilm dispersal using DNase enzymes may be appropriate for *B*. *pseudomallei* biofilm control.

## Materials and methods

### Ethics statement

*Burkholderia pseudomallei* B1, P1 and L1 clinical isolates were from the Melioidosis Research Center, Khon Kaen University (MRC, KKU). Clinical isolates used were not specifically isolated for this study but had been collected as part of a previous study of the epidemiology of *B*. *pseudomallei* in Khon Kaen Province. Patients cannot be identified: the isolates were anonymous and de-identified when we received them. Approval for the study was given by the Khon Kaen University Ethics Committee for Human Research (HE490324).

### Bacterial strains and growth conditions

*B*. *pseudomallei* isolates used in this study are listed in Table 1. Each bacterial isolate from glycerol stock at -80°C was grown on Ashdown’s agar and incubated at 37°C for 48 h. A single colony of *B*. *pseudomallei* was inoculated into 3 mL of Luria-Bertani (LB) broth containing appropriate antibiotics and incubated at 37°C with shaking (200 rpm) for 18 h. A 2% inoculum was added into 25 mL fresh LB broth and further incubated until OD_540_ = 0.8–0.9 to provide bacterial starter culture [27, 31, 32].

**Table 1:** Bacterial strains used in this study.

Bacterial strains	Characteristics	Sources/Description	References
B1	Clinical isolate	Blood	MRC, KKU
L1	Clinical isolate	Lung	MRC, KKU
P1	Clinical isolate	Pus	MRC, KKU
3E	Environmental isolate	Soil, Northeastern Thailand	MRC, KKU
8E	Environmental isolate	Soil, Northeastern Thailand	MRC, KKU
23E	Environmental isolate	Soil, Northeastern Thailand	MRC, KKU
ST39	Environmental isolate	Soil, Northeastern Thailand	[32]
H777	Clinical isolate, moderate biofilm-producing wild type	Blood	[31]
M10	Biofilm mutant strain of H777	M10 was constructed by the transposon mutagenesis to inactivate *bpsl0618*, a putative sugar transferase gene.	[31]
C17	Biofilm complemented strain of M10	C17 was constructed by restoring the *bpsl0618* gene in M10.	[27]

### Quantification of biofilm and eDNA in biofilms in 96-well plates

Two-day biofilm formation of *B*. *pseudomallei* was determined by crystal violet staining in 96-well microtiter plates as previously described by Taweechaisupapong et al, 2005 and Kunyanee et al, 2016 [27, 31]. Two hundred microliters of starter culture was dispensed, in replicates of eight, into each well of a 96-well flat-bottomed polystyrene plate (Nunclon #167008, Thermo Scientific, Denmark) and incubated at 37°C for 3 h to allow bacterial adhesion. Negative controls containing no *B*. *pseudomallei* were included. Following incubation, non-adhering bacteria were removed, then fresh LB medium was added and plates further incubated for another 21 h. Non-adhering bacteria were again removed before the biofilms were carefully washed with sterile distilled water and the wells refilled with fresh LB medium. After incubation for an additional 24 h, biofilms were carefully washed three times with sterile distilled water. The 2-day biofilm in each well was fixed with 99% methanol for 15 min and air dried. The biofilms were stained with 2% w/v crystal violet for 5 min. The excess stain was removed with running tap water. After air-drying, adherent crystal violet stain was dissolved in 200 μL 33% (v/v) glacial acetic acid and the optical density at 620 nm of each sample was measured by a microplate reader (TECAN Safire, Port Melbourne, Australia).

The quantity of eDNA associated with *B*. *pseudomallei* biofilm was examined in 96-well black plates (SPL Life Sciences, Korea) in triplicates concurrent with the biofilm quantification. The 2-day biofilm culture was rinsed three times with sterile distilled water. eDNA in each well was mixed with 200 μL of freshly prepared QuantiFluor dsDNA dye in TE buffer for 5 min (QuantiFluor dsDNA System, Promega, Madison, WI, USA) before measuring the fluorescence intensity (excitation 504 nm/emission 531 nm) using a fluorometer (Varioskan Flash Multimode Reader, Singapore) with SkanIt Software 2.4.3 RE for Varioskan Flash. Lambda DNA (QuantiFluor dsDNA System) was used to generate a standard curve for each run.

### Confocal laser scanning microscope observation

*Burkholderia pseudomallei* biofilm architecture and quantity of eDNA associated with the biofilm were evaluated on glass coverslips immersed in bacterial culture in 24-well plates (Costar #3524, Corning, NY, USA) using an Amsterdam Active Attachment (AAA) model slightly modified from previous descriptions [27, 33–35]. In brief, the set of 12 mm-diameter round glass coverslips attached to the sterile AAA model’s lid were autoclaved. The glass coverslips held on the lid were immersed into 1 mL of bacterial starter culture in each well of a 24-well plate and incubated at 37°C for 3 h. The lid was then transferred to a new 24-well plate containing fresh LB medium and incubated for another 21 h. The adhered bacteria on the coverslips were subsequently washed with sterile distilled water before being submerged into fresh LB medium and incubated for another 24 h to produce a 2-day biofilm. Three-hour, 24-h and 2-day biofilms on the glass coverslips were rinsed three times with sterile distilled water prior to staining with 50 μg/mL fluorescein isothiocyanate-concanavalin A (FITC-Con A) (Sigma, Saint Louis, Missouri, USA), which binds extracellular polysaccharide (representing biofilms) and 2 μM TOTO-3 (Thermo fisher Scientific, Oregon, USA), which stains eDNA present within biofilm, for 20 min according to the manufacturers’ instructions. Bacterial viability within 2-day biofilms was examined after staining with 3.34 μM SYTO 9 (live cells) and 5 μg/mL propidium iodide (PI) (dead cells) (Invitrogen, Thermo fisher Scientific, Oregon, USA) for 15 min. Stained biofilms were subsequently fixed with 2.5% glutaraldehyde for 3 h before being washed three times with sterile water and air-dried. The structure of the stained biofilm, eDNA present and bacterial viability on coverslips were visualized by confocal laser scanning microscope (CLSM, LSM 800, Carl Zeiss, Jena, Germany). The biofilm intensity was analyzed from z-stack processing using Zen blue software [13, 27]. Biomass of adherent cells and eDNA quantity were calculated with the COMSTAT computer program [36]. The bacterial viability is presented as live/dead ratio.

### DNase I treatment of *B*. *pseudomallei* biofilms and addition of exogenous DNA to biofilms

The role of eDNA in *B*. *pseudomallei* biofilm formation was investigated by depletion of eDNA using DNase I (Roche, Mannheim, Germany). DNase at a final concentration of 0.01, 0.1 or 1 U/mL was used, following Kim et al [37] with slight modification. DNase I was added into bacterial cultures at different time points representing various steps of biofilm development: 0 h (initial attachment), 24 h (biofilm formation) and 45 h (biofilm maturation). The selected DNase I concentrations were constantly maintained in the medium for up to 48 h at 37°C before the 2-day biofilms and eDNA were quantified by crystal violet staining and the QuantiFluor dsDNA System kit, respectively, and images obtained using CLSM. Untreated controls using LB media were also used for direct comparison.

Chromosomal DNA of *B*. *pseudomallei* H777 was extracted using a popular method described by Sambrook et al [38]. The DNA pellet was re-suspended with Tris-HCl buffer. Exogenous DNA, namely chromosomal DNA of *B*. *pseudomallei* or salmon-sperm DNA (Sigma, St Louis, MO, USA) (0.1 μg/mL), was added either simultaneously to the starting culture or the DNase-treated biofilm after removal of DNase by washing twice with sterile distilled water. DNase I enzyme, DNase I buffer and Tris-HCl buffer (diluent of the exogenous DNA) were also used as controls.

### Statistical analysis

Statistical analyses were performed using SPSS software, version 23 (SPSS Inc., Chicago, IL, USA). Biofilm formation and eDNA quantities produced by clinical and environmental isolates were analyzed using the nonparametric Kruskal-Wallis test followed by Dunn’s post-hoc test for comparison between pairs. Pearson correlation analysis of equal variance data was used to determine the relationship between biofilm biomass and eDNA quantity. Biofilm, eDNA and live/dead ratios were analyzed for statistical significance using the one-way ANOVA followed by Tukey’s post-hoc test, or the Games-Howell post-hoc test to correct for variance heterogeneity. Variance heterogeneity was assessed by Levene's tests. The levels required for statistical significance were * p < 0.05 and ** *p* < 0.001.

## Results

### Variations of biofilm formation and eDNA quantities of *B*. *pseudomallei*

**Fig 1:**
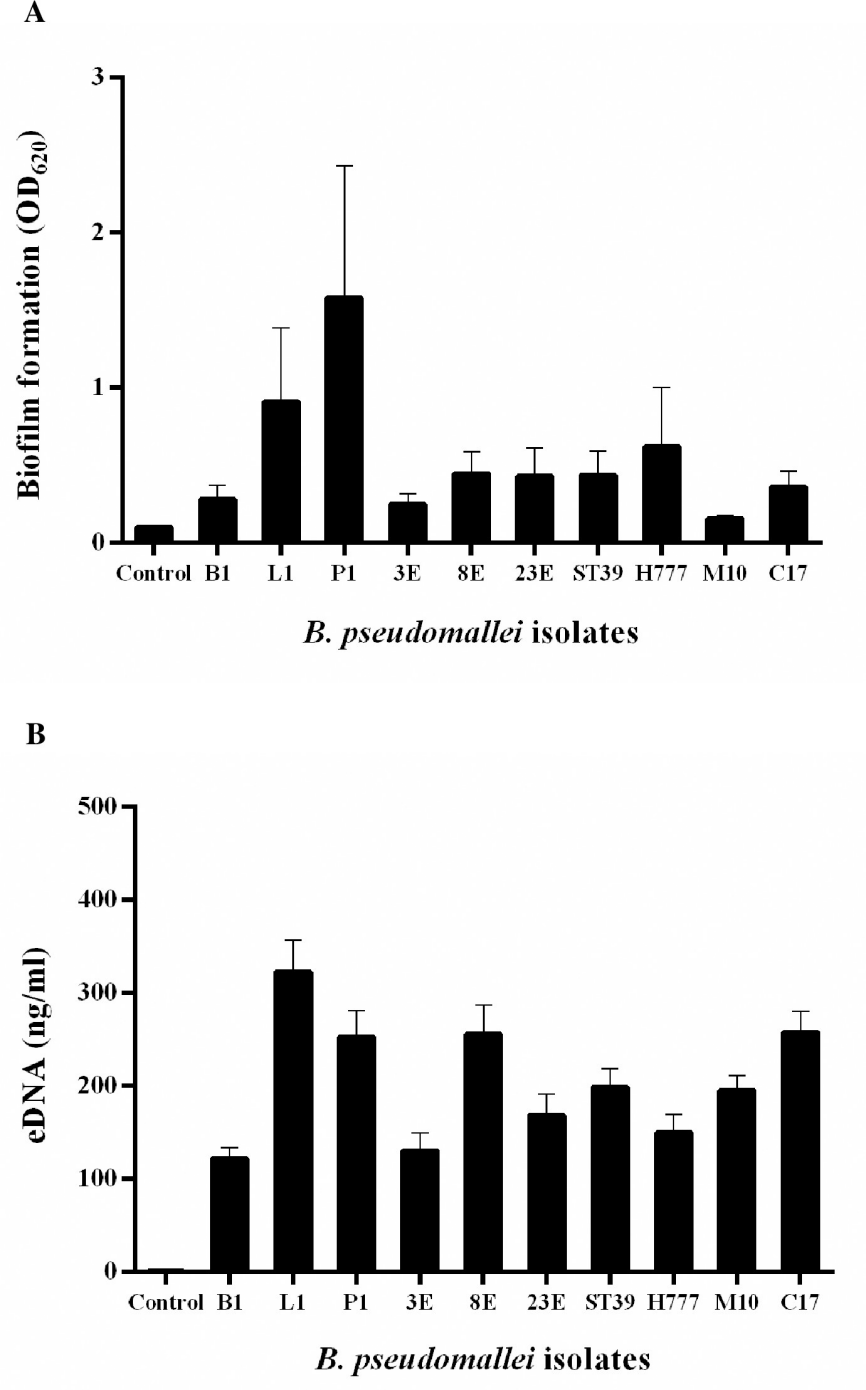
Variation in *B*. *pseudomallei* biofilm formation and eDNA quantity. (A) Degree of biofilm formation by 10 *B*. *pseudomallei* isolates grown in LB in 96-well plates at 37°C for 2 days was assessed using crystal-violet absorbance (OD_620_). (B) eDNA concentration in 2-day biofilm of 10 *B*. *pseudomallei* isolates was assessed using the QuantiFluor dsDNA System. Controls were LB medium lacking bacteria. Data are represented as mean ± SD from at least three independent experiments.

The static 2-day biofilm and eDNA associated with that biofilm of 10 different *B*. *pseudomallei* isolates (Table 1), stained with crystal violet and QuantiFluor dsDNA reagent, demonstrated the variation of biofilm formation (Fig 1A) and eDNA quantity (Fig 1B). Included were 4 clinical isolates (B1, L1, P1 and H777), 4 environmental isolates (3E, 8E, 23E and ST39), the biofilm mutant of H777 (M10) and the biofilm complemented strain of M10 (C17) in LB medium. Notably, P1 (isolated from pus) exhibited the greatest ability to form biofilm followed by L1 (from lung) and H777 (from blood) whilst L1 produced the most eDNA. Strains isolated from lung (L1) and pus (P1) produced significantly more biofilm than did blood isolates (B1 and H777) (*p* < 0.05) and all environmental isolates (*p* < 0.001).

### eDNA localized in *B*. *pseudomallei* biofilm and correlated with biofilm formation

**Fig 2:**
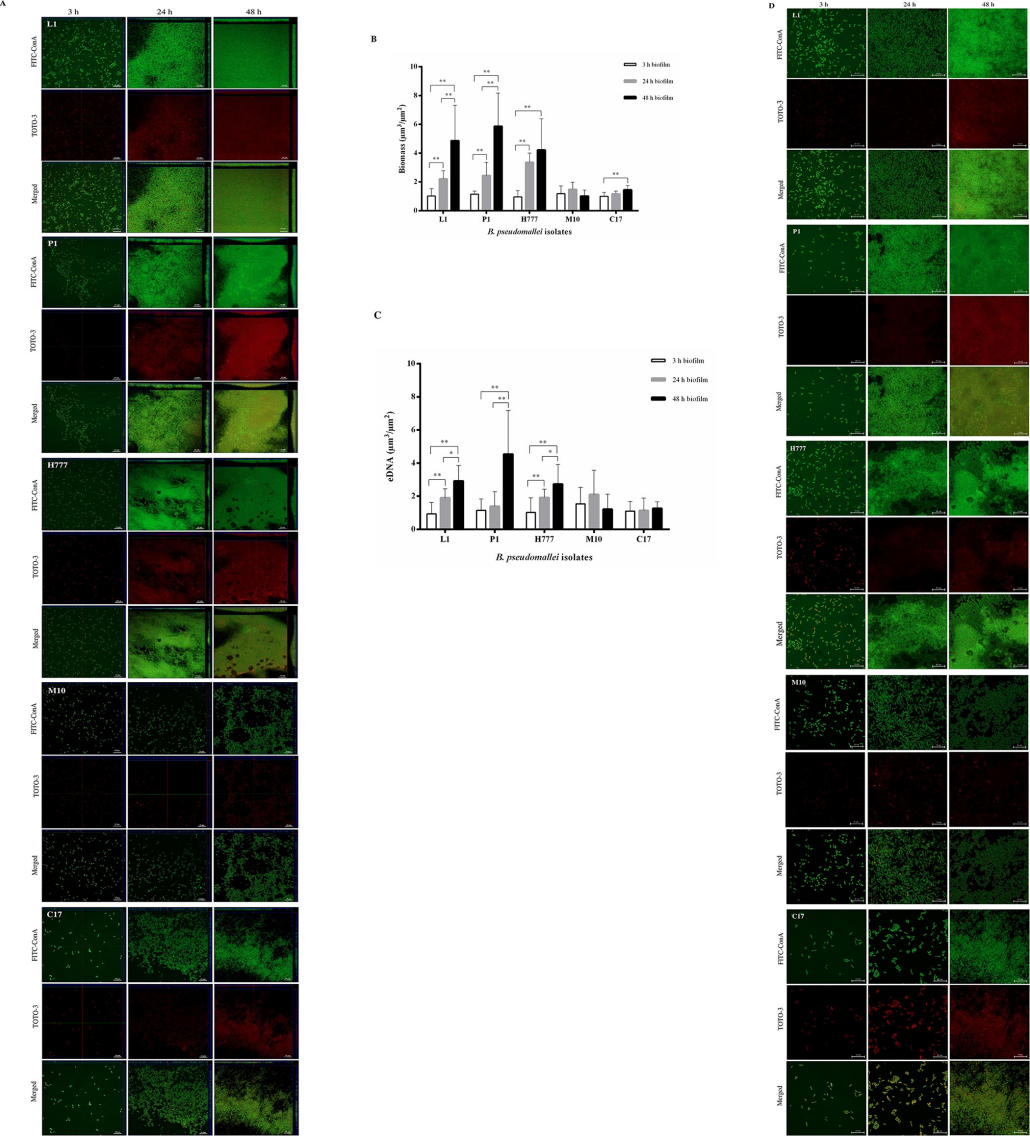
Biofilm and eDNA production by *B*. *pseudomallei* L1, P1, H777, M10 and C17 strains at 3, 24 and 48 h. (A) CLSM images of *B*. *pseudomallei* L1, P1, H777, M10 and C17 biofilms grown statically on glass cover slips in LB broth, 37°C at 3, 24 and 48 h after staining with FITC-ConA (biofilm, green) and TOTO-3 (eDNA, red). These CLSM images are representatives of three independent experiments. The images were taken under a Zeiss 800 CLSM microscope (63 × magnification). Scale bar represents 10 μm. (B and C) COMSTAT image analysis at 3 (white bars), 24 (grey bars) or 48 h (filled bars) indicating biofilm biomass and eDNA quantity. Data from 18 images (6 image z-stacks from three independent experiments) was used in each analysis. Significance was determined by one-way ANOVA, compared to 3 h biofilm development. * p < 0.05 and ** *p* < 0.001. (D) CLSM 2D images (100× magnification) of *B*. *pseudomallei* L1, P1, H777, M10 and C17 biofilms at 3, 24 and 48 h stained with FITC-ConA (biofilm, green) and TOTO-3 (eDNA, diffuse red or dead cells, red). TOTO-3-stained eDNA is present diffusely in and around *B*. *pseudomallei* biofilm. Scale bar: 10 μm.

The clinical *B*. *pseudomallei* isolates (L1, P1, H777); the biofilm mutant (M10) and the biofilm complement strains (C17) were chosen for further investigation. We examined the biofilm architecture and eDNA quantity on glass coverslips after staining with FITC-ConA and TOTO-3 using CLSM. FITC-ConA stains biofilm green, whereas TOTO-3 stains eDNA red (Fig 2A). We observed an increase in adhered cells as well as eDNA signal intensities on the glass coverslips over time as biofilm developed. Moreover, the CLSM images revealed morphological differences of biofilm and eDNA signal intensities among isolates (Fig 2A) which are in line with our initial findings of variation in biofilm biomass and eDNA production (Fig 1). The biofilm complement strain, C17, produced a flattened biofilm architecture which covered the coverslips but the wildtype biofilm phenotype was not fully restored. The biofilm mutant failed to form the tower structures of mature biofilm. CLSM images clearly demonstrated the localization of eDNA within the biofilm matrix of *B*. *pseudomallei*. The highest red fluorescence of eDNA was seen in the wild-type isolates (L1, P1 and H777), especially in the 48 h biofilm.

To further investigate the hypothesis that eDNA is important for development of biofilm, we also analyzed the Z-stack confocal images with the COMSTAT image-analysis software. This revealed significant increase of biofilm biomass and of eDNA concentration in biofilm over time in several strains: the biofilm wild-type *B*. *pseudomallei* L1, P1 and H777 (Fig 2B and 2C). Pearson correlation analysis demonstrated a significant positive correlation in these strains between eDNA production in biofilm and biofilm biomass (*p* < 0.001) (Table 2). Higher magnification CLSM 2D images (100×) clearly demonstrated diffuse eDNA surrounding bacterial biofilms, emphasizing the extracellular location of this DNA (Fig 2D).

**Table 2:** Pearson correlation analysis between biofilm biomass of *B*. *pseudomallei* and quantity of eDNA.

eDNA	Biofilm biomass of *B*. *pseudomallei*				
	L1	P1	H777	M10	C17
Pearson’s correlation coefficient	0.741	0.914	0.788	-0.232	-0.085
*p* value	< 0.001	< 0.001	< 0.001	0.091	0.540

**Fig 3:**
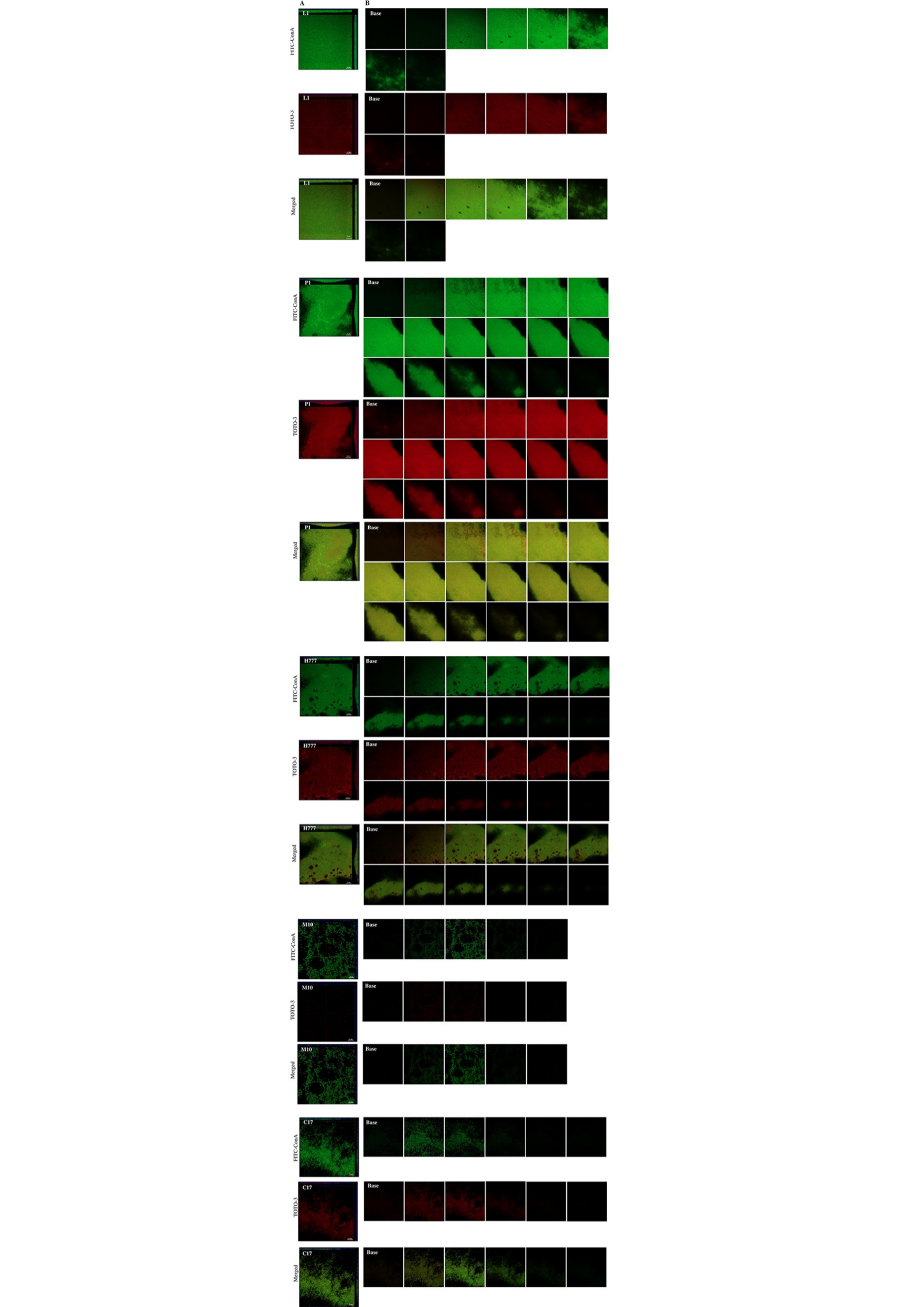
*B*. *pseudomallei* L1, P1, H777, M10 and C17 biofilm structure. (A) CLSM images of biofilm and eDNA of *B*. *pseudomallei* grown statically on glass slides in LB broth at 37°C then stained with FITC-ConA (biofilm, green) and TOTO-3 (eDNA, red). The images are representative of three independent experiments and were acquired using a Zeiss 800 CLSM microscope (63×magnification). (B) Serial-section gallery of 2-day FITC-ConA/TOTO-3-stained biofilms with an increment of 1 μm on the Z-axis (reconstructed in Fig 3A).

Biofilms of strains L1, P1, H777, M10 and C17 varied greatly in thickness according to the serial-section gallery of the 2-day FITC-ConA/TOTO-3-stained biofilms (Fig 3A and 3B). The thickest biofilms and the greatest quantity of eDNA were produced by strain P1, followed by L1, H777 and C17. In addition, the relative slice gallery revealed eDNA particularly at the base of the biofilm, as indicated by red fluorescence on the bottom of the images, while the upper layer displayed a predominance of green cells.

### eDNA released from living *B*. *pseudomallei* cells

**Fig 4:**
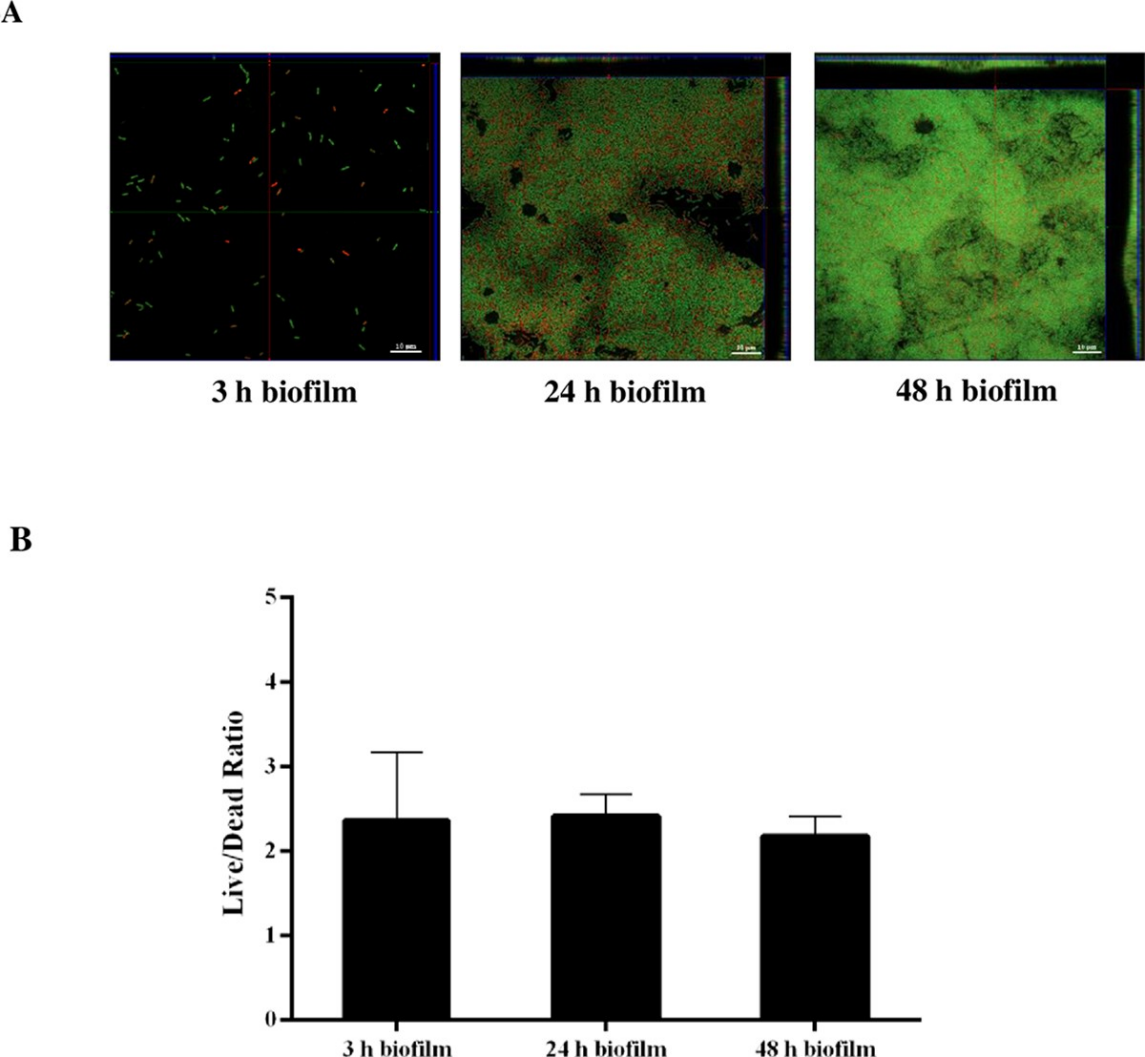
Live/dead staining and live/dead ratio of cells in *B*. *pseudomallei* H777 biofilm at 3, 24 and 48 h. (A) Live/dead staining of *B*. *pseudomallei* H777 biofilms at 3, 24 and 48 h. The scale bar represents 10 μm. The images were taken using a Zeiss 800 CLSM microscope (63× magnification). (B) COMSTAT image analysis of live/dead ratio of *B*. *pseudomallei* H777 biofilms at 3, 24 and 48 h. The graph presents the mean ± SD from 18 CLSM micrographs in 3 independent experiments.

We determined whether eDNA was released during biofilm formation primarily from living or dead bacterial cells. To do this, live/dead staining of the 3, 24 and 48 h *B*. *pseudomallei* H777 biofilm was employed. CLSM imaging and COMSTAT analysis revealed a constant live/dead ratio during the observation period (Fig 4A and 4B). Given that eDNA accumulates through time in biofilm, this implies the liberation of eDNA from living bacterial cells.

### DNase I reduces *B*. *pseudomallei* biofilm

**Fig 5:**
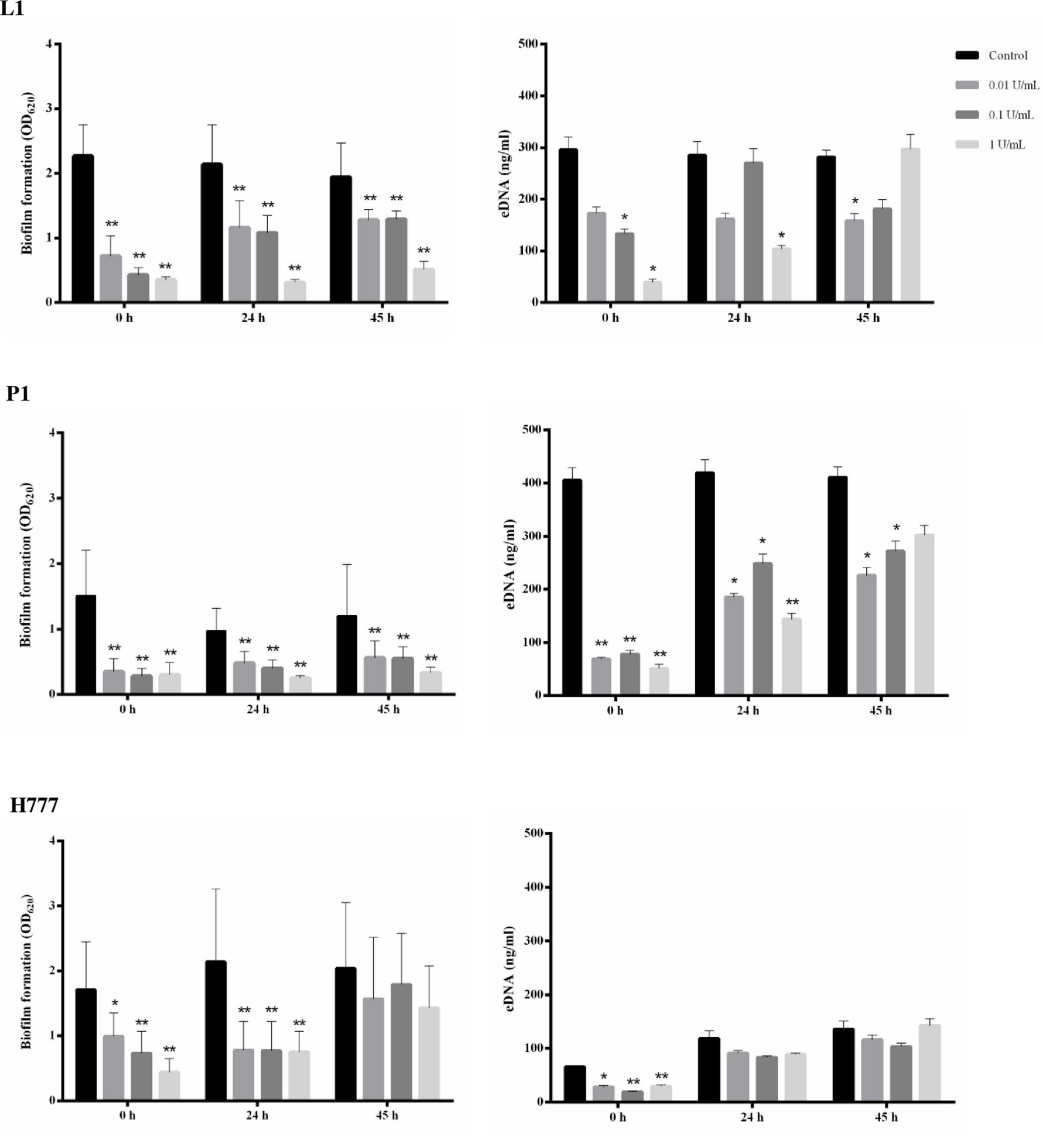
Impact of DNase I treatment on *B*. *pseudomallei* biofilm formation. Static biofilms of *B*. *pseudomallei* strains L1, P1 and H777 in LB were treated with DNase I (0.01, 0.1 and 1 U/mL) at 0 h, 24 h or 45 h after inoculation and maintained for up to 48 h. Biofilm formation and eDNA concentration of the 2-day biofilms were assessed using crystal-violet absorbance (OD_620_) and the QuantiFluor dsDNA System, respectively. DNase I buffer acted as control. Biofilm formation of each strain was examined in eight replicates and eDNA was quantified in duplicates, on three independent occasions. Data represents mean ± SD. * *p* < 0.05, ** *p* < 0.001.

The impact of DNase I on biofilm formation by *B*. *pseudomallei* at different time points was investigated in microtiter plates followed by crystal-violet staining for biofilm and fluorescence intensity for the eDNA associated with the biofilm. DNase I (0.01, 0.1, and 1 U/mL) was added to the *B*. *pseudomallei* biofilm culture in LB at 0, 24 and 45 h, and maintained in the culture for up to 48 h. Biofilms of *B*. *pseudomallei* strains L1, P1 and H777 were considerably reduced when DNase I had been present in the bacterial culture since initial adhesion (0 h) and biofilm formation (24 h) stages compared with the untreated controls (* *p* < 0.05 and ** *p* < 0.001) (Fig 5). These results emphasize the role of eDNA in initial adhesion and biofilm formation stages. When DNase I was added at the 45 h preformed-biofilm stage, biofilm was reduced in the L1 and P1 strains, but not in the H777 strain (Fig 5). DNase I noticeably lowered eDNA concentrations in biofilm if the enzyme was added into the starting inoculum (0 h). The remaining eDNA at the 24 h and 45 h preformed biofilm stages may have been released from dispersed bacterial cells in the wells.

**Fig 6:**
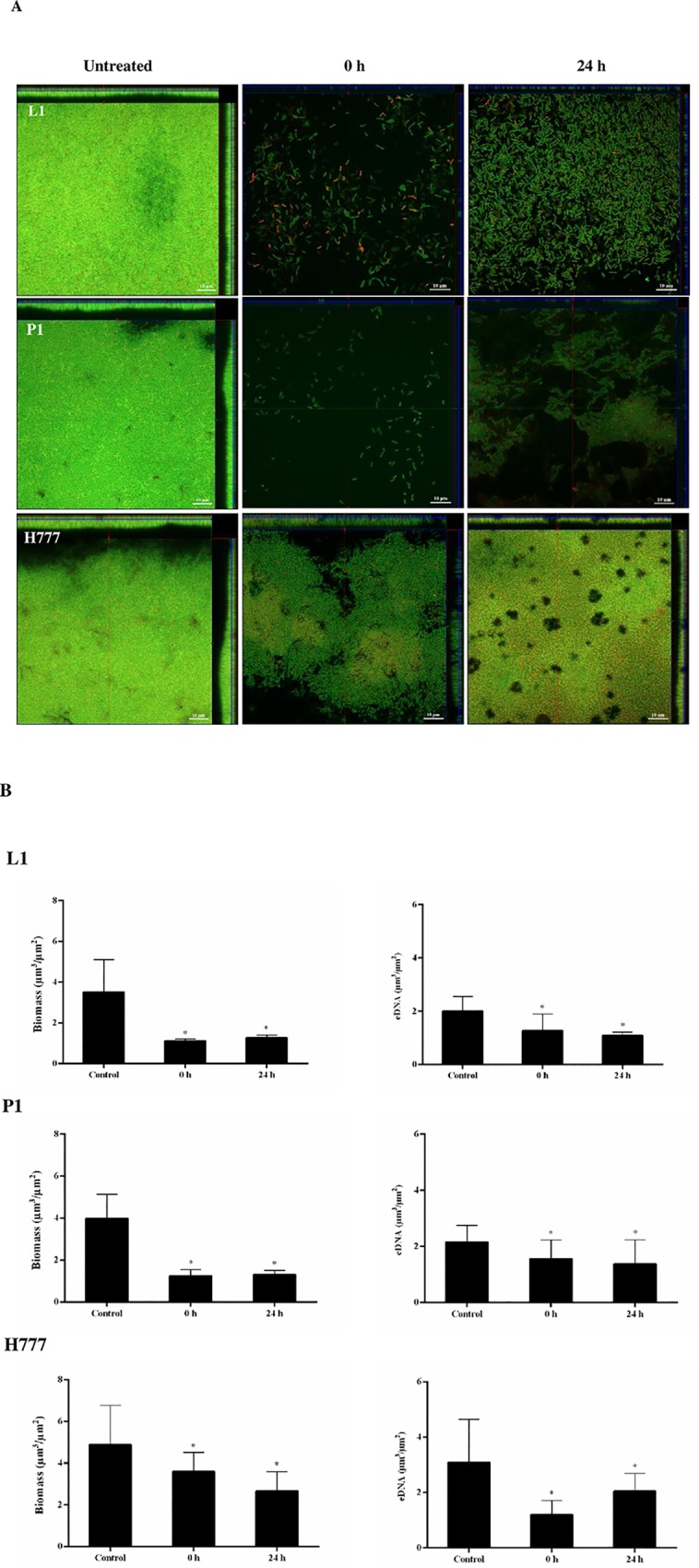
DNase I treatment affects initial attachment and biofilm formation of *B*. *pseudomallei*. *B*. *pseudomallei* L1, P1 and H777 biofilms were grown in LB at 37°C. The biofilms were treated with DNase I (0.01 U/mL) at 0 h and 24 h post-seeding and maintained until 48 h. (A) CLSM images of DNase I treated biofilm structure and eDNA on coverslips. The 2-day biofilm architecture and quantity of eDNA were examined after staining with FITC-ConA (green) and TOTO-3 (red), respectively. The scale bars indicate 10 μm. The images were taken using a Zeiss 800 CLSM microscope (63× magnification). (B) COMSTAT image analysis of DNase I-treated *B*. *pseudomallei* biofilms and eDNA. Data represents mean ± SD of 18 images from three independent experiments. * *p* < 0.05.

The effects of 0.01 U/mL DNase I on initial attachment and biofilm-formation stages were further witnessed using CLSM after staining with FITC-ConA and TOTO-3. The CLSM images showed that continuous presence of the enzyme from the starting inoculum (0 h) or 24 h (Fig 6) until the end of the experiment at 48 h clearly diminished biofilm formation of all tested *B*. *pseudomallei* strains relative to controls (Fig 6A). Biofilms treated with DNase I appeared thinner than in untreated controls. Statistical analysis of images showing DNase I-treated biofilm and eDNA at 0 and 24 h using the COMSTAT software confirmed the suppressive effect of the enzyme on initial adhesion (0 h) and biofilm formation (24 h) compared to untreated controls (* *p* < 0.05) (Fig 6B). This suggests that DNase I downgrades *B*. *pseudomallei* biofilm development, in particular during initial biofilm formation.

### Exogenous chromosomal DNA did not alter *B*. *pseudomallei* biofilm formation pattern

**Fig 7:**
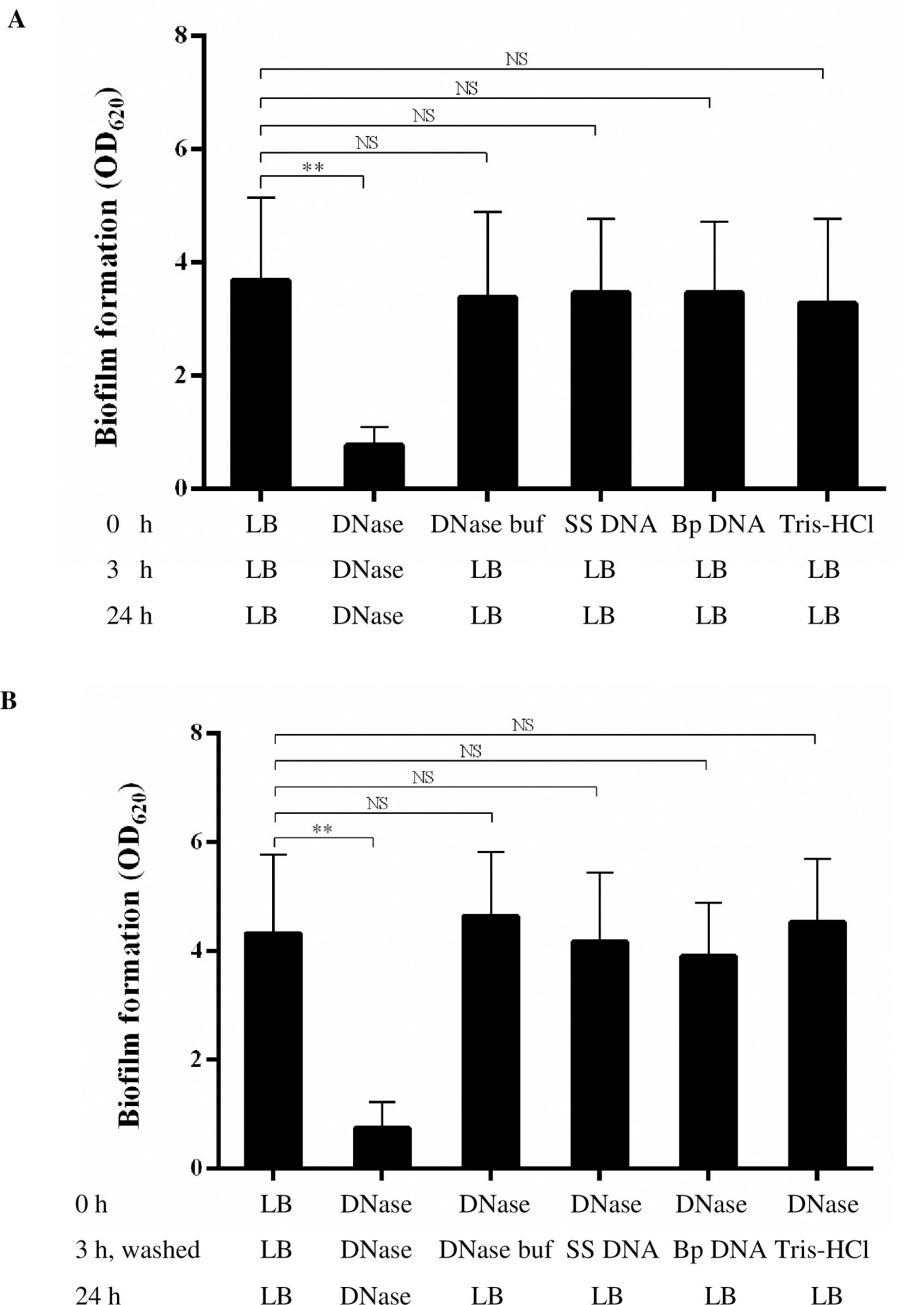
Exogenous chromosomal DNA did not alter either untreated biofilm or DNase I-treated biofilm of *B*. *pseudomallei* H777. (A) Amount of 2-day *B*. *pseudomallei* H777 biofilm formed in LB, treated with DNase I, supplemented with either salmon sperm DNA (SS DNA) or *B*. *pseudomallei* genomic DNA (Bp DNA) compared to the controls. Data represents mean ± SD from three independent experiments. (B) Amount of 2-day *B*. *pseudomallei* H777 biofilm formed in LB after treatment with 0.01 U/mL DNase I for 3 h, followed by washing steps to remove DNase, and then supplemented with exogenous salmon sperm DNA or *B*. *pseudomallei* genomic DNA. Data represents mean ± SD from three independent experiments. ** *p* < 0.001, NS = non-significant.

Given the association of eDNA with the biofilm matrix and the sensitivity of biofilm development to DNase, we further questioned whether exogenous DNA may raise *B*. *pseudomallei* biomass. Therefore exogenous chromosomal DNA of *B*. *pseudomallei* and salmon sperm DNA were added into biofilm cultures of *B*. *pseudomallei* H777, either at 0 h or after eDNA depletion using DNase I followed by washing steps to remove DNase I. The results demonstrated that exogenous DNA could not alter biofilm-formation ability of *B*. *pseudomallei* either in normal conditions or after DNase treatment (Fig 7A and 7B). Notably, structure of DNase-treated biofilm was spontaneously rebuilt without any addition of exogenous DNA after removal of the enzyme. These data may indicate that eDNA released from *B*. *pseudomallei* cells present at the time is adequate for biofilm reconstruction.

## Discussion

Growing as a biofilm contributes not only to the survival of bacterial cells in unfavorable environments but also shields them from antimicrobial agents and host immune defenses. Numerous attempts have therefore been made to find effective means of biofilm dispersal or to prevent biofilm formation, thus increasing susceptibility of bacterial pathogens to antimicrobial agents and host defenses. For that reason, biofilm composition, mechanisms of formation and structure need to be understood. One of the key biofilm components is eDNA, which essential for biofilm development during bacterial adhesion and provides structural support for biofilm formation in both Gram-negative and Gram-positive bacteria [5, 7, 10, 37, 39–41]. Biofilm of *B*. *pseudomallei* is known to act as a barrier for antimicrobial agents and is associated with relapsing melioidosis [21, 24]. It has not yet been established whether eDNA plays a role during *B*. *pseudomallei* biofilm formation. The knowledge from this study indicates that eDNA can be a primary target for eradication strategies against *B*. *pseudomallei* biofilm.

In this study, we demonstrated variations in biofilm formation and eDNA quantities among clinical and environmental *B*. *pseudomallei* isolates by staining with crystal violet and the QuantiFluor dsDNA System. Similar variation in biofilm-forming capacity among 50 strains of *B*. *pseudomallei* was previously reported by Taweechaisupapong [31]. Clinical isolates of *B*. *pseudomallei* from lung and pus showed greater ability to form biofilm, in line with the higher eDNA amounts produced. However, eDNA levels were unpredictable in strains less capable of biofilm formation, including the biofilm mutant, M10. CLSM was performed for further quantitative and qualitative investigation of biofilm biomass and eDNA. CLSM micrographs of *B*. *pseudomallei* biofilm on coverslips stained with FITC-ConA (biofilm) and TOTO-3 (eDNA) demonstrated the different *B*. *pseudomallei* biofilm architectures among isolates. The eDNA was shown to be present primarily at the base of the matrix of *B*. *pseudomallei* biofilm. These findings hint at the participation of eDNA in initial steps of biofilm formation. Visualization and quantification of biofilms and eDNA using CLSM when isolates were grown on coverslips for visualization and quantitative analysis using CLSM gave more consistent data than did crystal violet staining and fluorescence detection in wells. CLSM images also provided direct evidence of eDNA in biofilm structure, indicating that eDNA is essential for biofilm formation. In addition, we have provided the first report of significant positive correlation of *B*. *pseudomallei* biofilm biomass with eDNA quantity, emphasizing the contribution of eDNA to biofilm formation.

The drastic increase of eDNA quantity through time (Fig 2C) despite the constant and high live/dead ratios of bacterial cells in 3, 24 and 48 h biofilm (Fig 4) points toward the liberation of eDNA from living *B*. *pseudomallei*. This is consistent with a previous report that demonstrated accumulation of *B*. *pseudomallei* eDNA on murine gastric tissues without bacterial cell lysis [28]. Mechanisms of liberation of eDNA from *B*. *pseudomallei* cells remain to be elucidated: knowledge of these might help in devising strategies to counteract biofilm formation by this pathogen.

CSLM images and the COMSTAT analysis demonstrated that depletion of eDNA by DNase I treatment considerably reduced biofilm formation, depending on the biofilm stage at which DNase treatment was started. The presence of DNase in biofilm culture from 0 h or 24 h, with maintenance of DNase I in the cultures up to the end of the experiment at 48 h, significantly reduced *B*. *pseudomallei* biofilm. This emphasizes the importance of eDNA as an intercellular connector during initial attachment and early biofilm development. However, DNase intervention after biofilm has reached the mature stage of development led to reduction of eDNA in only 2 of 3 clinical *B*. *pseudomallei* isolates. It is possible that the DNase could not gain access to eDNA in the mature biofilm matrix [10, 42]. Our findings are consistent with previous reports that demonstrated the essential role of eDNA during the initial step of biofilm formation by other bacteria. Harmsen and colleagues demonstrated, by use of DNase I, that eDNA was involved in the initial attachment of *Listeria monocytogenes* biofilm [15]. Kim and colleagues demonstrated DNase I can inhibit the initial step of biofilm formation of *Campylobacter* strains isolated from raw chicken [37]. Several reports have demonstrated that DNase I can inhibit the biofilm formation of *Pseudomonas aeruginosa* [10, 43], *Escherichia coli*, *Staphylococcus aureus* [44], *Campylobacter* sp. [37], *Xanthomonas citri* subsp. *citri* [8], *Neisseria meningitidis* [15] and *S*. *epidermidis* [45]. Svensson and colleagues demonstrated DNase I treatment decreased the biofilm formation and stress tolerance of *C*. *jejuni* [46]. Mann and colleagues demonstrated that DNase I inhibits biofilm formation and biofilm maturation in *Staphylococcus aureus* [13] In addition, Kim and colleagues demonstrated that DNase I significantly inhibits the mature biofilm of *Campylobacter* strains when treated with DNase I after 72 h of biofilm formation [37]. However, Whitchurch and colleagues demonstrated that DNase I could not abolish 84 h *P*. *aeruginosa* biofilm [10]. Similar findings for *Helicobacter pylori* suggest that eDNA may not be the main component of *H*. *pylori* biofilm [12].

To our knowledge, there are two previous reports about eDNA of *Burkholderia* species. Austin and colleagues demonstrated that *B*. *pseudomallei* strain K96243 produced eDNA without bacterial cell lysis and that eDNA enhanced bacterial colonization of stomach tissues of BALB/c mice [28]. Meanwhile, Garcia and colleagues revealed that eDNA is not required for the initial attachment step but essential for cellular interaction of 16 h biofilm formation in *B*. *thailandensis* [29].

The addition of exogenous *B*. *pseudomallei* genomic DNA and salmon-sperm DNA during biofilm inoculation, prior to or after DNase I treatment, did not produce a detectable effect on *B*. *pseudomallei* biofilms. This is in contrast with observations by Harmsen and colleagues, who demonstrated that the addition of genomic DNA and salmon-sperm DNA restored the biofilm of *L*. *monocytogenes* at the initial attachment stage, indicating that eDNA is required for the adhesion step of biofilm formation in that species [15]. Similarly, Carrolo and colleagues demonstrated that the addition of salmon-sperm DNA could restore the ability of mutant strains of *Streptococcus pneumoniae* to form biofilms [47].

In conclusion, our findings may have important biological implications by pointing out that eDNA is a key component of *B*. *pseudomallei* biofilm, in particular during the early stages of biofilm growth. eDNA could be an attractive target for prevention of *B*. *pseudomallei* biofilm formation by using DNase digestion. This points the way to novel strategies to destabilize *B*. *pseudomallei* biofilm formation: perhaps a combination of DNase I and antibiotics will lead to an effective treatment for relapsing melioidosis.
